# Ten Simple Rules To Commercialize Scientific Research

**DOI:** 10.1371/journal.pcbi.1002712

**Published:** 2012-09-27

**Authors:** Anthony C. Fletcher, Philip E. Bourne

**Affiliations:** 1 Salix Management Consultants Ltd, London, England; 2 Department of Pharmacology, University of California San Diego, La Jolla, California, United States of America; 3 Skaggs School of Pharmacy and Pharmaceutical Sciences, University of California San Diego, La Jolla, California, United States of America

Commercializing scientific research or a breakthrough idea is really no different, in principle, from commercializing anything, except perhaps that it's more difficult in practice because of the steps required to turn basic research into something practical and because you are looking for a market for a product, rather than designing a product to fit an established, or obvious market.

Commercialization is different to starting and running a company, a broader endeavour and the subject of a previous Ten Simple Rules article [1]. Even so, commercialization can be a broad endeavour. For example, at one extreme, you could hand over your monoclonal antibody to Sigma to supply it on your behalf to other researchers who might find it useful while the company pays you a small royalty; on the other, you could be involved in developing Herceptin (anti-HER2 monoclonal antibody) from its origins as a mouse-specific antibody through to its use as an effective anti-breast cancer drug, in a process that took more than decade. Here we assume the former—others are carrying out that commercialization, which has its pluses and minuses—less work for you, but typically less control of the commercialization process.

Commercialization is a much studied subject, both by academics [2] and the business community [3]. All larger academic institutions generally have offices to promote and help scientists get research to market. Consequently, in this Ten Simple Rules article we won't deal with the details, but instead will concentrate on some of the key issues to consider when working with, or before and after working with, a specialized office.

## Rule 1: What Drives Science Does Not Drive Business

Scientists evaluate research by considering whether it makes an original contribution to our understanding of the world. Businesses have a different rationale, which, by and large, is to make money. This engenders a huge culture gap. In the 18th century, as the Chinese started to make porcelain for European markets, it was noted that they simply didn't get the idea of perspective. Pagodas appeared the size of flower vases. The artists understood symbolism; Europeans sought realism. And so it is with commercialization: scientists are not primed for business (some would even say this goes against academic freedom) and businesses are not, for the most part, so good at science unless they have specialized research divisions—Bell Labs comes to mind here, although these days an exception rather than a rule. When these worlds collide there is a need for intermediaries and translators to ensure a common understanding and successful path from research to commercialism. Scientists need to get business people who are “on the same wavelength” on their team and who can explain and guide them. Conversely, businesses have to be able to determine what research universities have to offer and how it could be of benefit. Interfaces are varied, ranging from university development offices to business outreach units to organizations like CONNECT (http://www.connect.org) that specialize on being the interface. These are valuable resources and should be utilized by both scientists and potential business partners.

## Rule 2: There Is No Single Path To Commercialization

Commercialization of scientific breakthroughs is something that has become more formalized in recent years thanks, in the United States at least, to the Bayh-Dole Act (legislation dealing with intellectual property arising from federal government–funded research) [4], with academia taking an active role in facilitating the translation of its intellectual capital into business. There are many routes for this: licensing, royalties, incubation, and in-house development. Industry itself has also moved physically closer to large universities (e.g. science parks) to share in the human capital. Beneath all this activity there are complex issues regarding how much potential value lies locked up in these intellectual assets and how they can best be developed without straying too far from the progenitors' ideals, and at the same time generating value. There are many ways to go from the laboratory bench to the store: commercialization is just like any business process– part art, part science; part inspiration, part perspiration. Most routes are essentially mechanistic, some work and some don't—there is no secret way to do things. So if anyone tells you at the start it's a sure fire winner (or not), don't believe them—there is a lot of hard work that has to be done to see if an idea can make it. And never believe advice that says “this is the best way” based on a single example—for every research-driven idea that makes it big, hundreds wither slowly away. These failures are hardly ever the subject of detailed case studies, and so we have no idea why they failed and what lessons we could learn.

## Rule 3: You Must Know Your Rights And Those Of Colleagues

This might sound obvious, but it is important to know who owns and who has the right to develop your research output. As academics, by default, most institutions (or less often, funders) own your research. The institution may choose to protect your ideas with copyrights, licenses, or patents, a wise idea if they are to have commercial value (see Rule 4). That protection is not on your behalf as the inventor, but on behalf of the institution(s) where the work was done. You need to understand what this protection means in terms of process, cost, and time involved. Research is collaborative, often with multiple institutions involved, and this can greatly complicate the rights and ownership of intellectual property. Such issues should have been thoroughly reviewed and agreed with all the relevant scientists before the research is disclosed. Good scientific collaborations can be ruined by misunderstood commercialization strategies.

## Rule 4: Consider The Implications Of Going From Public To Private

Academic research has many benefits, for example, collaboration, data and knowledge sharing, and freedom to publish. When moving this research into the private sector, different rules apply. There is a need to protect the intellectual property. In some cases, protecting that investment has implications for follow-on developments and impacts academic freedom. For example, consider a situation where a company licensing a technology from an academic institution also has the rights to follow-on developments. Those rights could impact the academic scientist's ability to freely publish those new developments.

## Rule 5: Decide How Much Of Yourself You Want To Give

At one extreme, you can give over your research completely and have little or nothing to do with subsequent commercialization; at the other extreme you could be heavily involved in the company commercializing your research or indeed found a company to develop the research. The level of engagement with the commercialization is going to define the time commitment and possibly financial reward coming from the commercialization. This needs to be thought about carefully at the outset and should be mapped to your longer-term career goals. Some academics want to, and do, make a successful transition to business—perhaps as happy heads of research and development (R&D), free from the administrative hassle, but a key part of the business—and some of course stay in academia. Markets have no sentiment and don't care what you do: they just care what you can contribute.

## Rule 6: Separate The R And The D And Be Realistic

There is a big difference between basic research and the development of such research to the point of commercialization. Generally, development is done by the entity commercializing the product and could be considered the mid-point between academic and commercial cultures. Development can be hugely expensive and time-consuming and presents a huge financial risk to the investor, especially as it is a front-loaded cost. The investor has to look at such topics as mass production (scaling up from lab levels), distribution, logistics, pricing, practicality, marketing, safety, the law, etc. Often times, one or more of these proves insoluble and the breakthrough has to languish, possibly for decades, until a solution appears. Personal genomics is an example where extensive commercialization of a number of ideas has had to wait until next generation sequencing makes the products feasible. Scientists also need to be realistic in valuing the idea—they typically have no concept of the development costs and often feel the basic research represents the bulk of the value, which is almost never the case.

## Rule 7: The Market May Not Exist At The Outset

The old fashioned method of working out what your factory can make (being “production led” in the jargon) and then seeing if there is a market is a largely discredited approach in modern business. In the case of basic scientific research, of course, this is exactly the situation—scientists invariably investigate things out of intellectual curiosity without any view to commercialization. The original research will not be aimed at solving any commercial, market-related problems, outside of obvious areas such as pharmaceuticals and engineering, and so the breakthrough is inevitably made in isolation of market requirements.

There are various anecdotes that illustrate the apparent lack of market. “Who needs music on the move?” was one comment about the Sony Walkman. “No one wants a tablet computer with no keyboard”, and so on. Examples like these are often used to “prove” that a good idea will make it anyhow, but it's simply not true in the majority of cases. It conveniently sidesteps the point that if no ready market exists, it has to be developed. That takes money, advertising, skill, and time. All of which add to the development costs.

## Rule 8: Consider The “Want” Versus The “Need”

There is a venerable marketing axiom that products should always address a need, not a want. People often express “wants”, but they buy “needs”. Consumers want a Ferrari but they buy a Toyota. It is so easy for an academic scientist to believe there is a need for a product resulting from their research when in fact it is a want (or to put it another way, it's a “nice to have” not a “must have”). Thus, commercialization of a breakthrough needs to address what people or other businesses will actually pay for—and this is a complex issue. Generally, a fair amount of time and money needs to be spent on market research to understand this—if people will not pay, then no matter how good the idea, it will never be successfully commercialized. Other market dynamics can also intervene: for example, a common issue is that of technologies that are never implemented because their payback time is greater than a market will bear. Market-related short-termism has killed many a promising idea.

## Rule 9: Make It Comprehensible

The people who are going to fund the development of your research and subsequently take it to market will be business people, not scientists, irrespective of whether the ultimate product is aimed at technical buyers. At the earliest stage you need to boil down the research into an “elevator pitch”—a few sentences the layperson can comprehend and one that sets out a clear reason to purchase. A mini reactor that fits in a suitcase and will power a domestic car for a year without recharging fits that model. A new aerogel does not—who knows what this does and what benefits it might confer? A common problem is that the relationship of the research to the final practical product may not be clear. One approach to solve this is by association: “Our breakthrough is a distinct improvement on…” Focus on the biggest profit opportunities in your early pitches. Business people prefer to see a clear track to a clear market opportunity rather than have to work it out for themselves.

## Rule 10: Customers Are The Ultimate Peer Review

As scientists, peer review of our research publications evaluates novelty, a correct and accurate scientific process, reproducibility, and value to the community. The example of Henri Poincaré is useful here to illustrate the value of peer review: the first version of his work on “The three-body problem” contained a serious error that was picked up during peer review. Alterations and changes then led to extremely important work on modern chaos theory. Consider also Frege's pioneering book on predicate logic at the turn of the century. Bertrand Russell read it in draft form and sent him a letter pointing out it was prey to Russell's Paradox (“the set of all sets not containing themselves…”), and Frege was able to add a note in proof acknowledging this and discussing ways out. In business, the analogy is the importance of testing out ideas and products before a full launch and then to listen carefully to what the ultimate consumers say. This market research is key; if the market is lukewarm, it doesn't matter how great the research, a product won't happen. You need to be prepared for the eventuality that while the market research does not indicate a product can arise as you envisioned, a different product might be possible. Is that what you want?

As we said at the outset, looking for a problem to fit your solution is always going to be tough going. And it's probably even tougher to find someone who will back you with money, time, and resources that will be needed to turn your scientific research into something that will benefit society. But don't give up. Post-it Notes were once a scientific curiosity, Teflon just flakes in a solution and penicillin contamination in a petri dish.

Do remember, however, that as the originating scientist, knowledge and recognition may be the only reward you get—others who take it to market (and take the financial and commercial risk) might get the majority of the money. But as an academic scientist, hopefully that's not why you entered science in the first place. There is increasing emphasis worldwide for making better practical use of fundamental scientific research from academia. Be part of the change.

**About The Authors**

**Anthony C. Fletcher** has a PhD in organic chemistry, and had a 20-year career as a management consultant with Deloitte and with Cap Gemini Ernst and Young and is now a freelance consultant.

**Philip E. Bourne** has a PhD in physical chemistry, is a professor of pharmacology and Associate Vice Chancellor for Innovation and Industrial Alliances at the University of California San Diego, the co-founder of SciVee.tv, and founding editor-in-chief of this journal. The authors have known each other since childhood and have been involved in one company together.

## References

[1] Fletcher AC, Bourne PE (2012) Ten simple rules for starting a company. PLoS Comput Biol 8: e1002439 doi:10.1371/journal.pcbi.1002439 22479171 10.1371/journal.pcbi.1002439 PMC3315446

[2] MIT Sloan School of Management (2012) Faculty & research: Fiona Murray. Available: http://goo.gl/tb1HB. Accessed 20 August 2012.

[3] Isis Innovation Ltd. Available: http://www.isis-innovation.com. Accessed 20 August 2012.

[4] Wikipedia contributors (2012) Bayh–Dole Act. Wikipedia, The Free Encyclopedia. Available: http://en.wikipedia.org/wiki/Bayh%E2%80%93Dole_Act. Accessed 20 August 2012.

